# Shiga Toxin Interaction with Human Intestinal Epithelium

**DOI:** 10.3390/toxins3060626

**Published:** 2011-06-14

**Authors:** Stephanie Schüller

**Affiliations:** Norwich Medical School, University of East Anglia, Norwich NR4 7TJ, UK; Email: stephanie.schuller@bbsrc.ac.uk; Tel.: +44-1603-255-282; Fax: +44-1603-255-288

**Keywords:** Shiga toxin, human intestinal epithelium, regulation, intracellular transport, translocation

## Abstract

After ingestion via contaminated food or water, enterohaemorrhagic *E. coli* colonises the intestinal mucosa and produces Shiga toxins (Stx). No Stx-specific secretion system has been described so far, and it is assumed that Stx are released into the gut lumen after bacterial lysis. Human intestinal epithelium does not express the Stx receptor Gb3 or other Stx binding sites, and it remains unknown how Stx cross the intestinal epithelial barrier and gain access to the systemic circulation. This review summarises current knowledge about the influence of the intestinal environment on Stx production and release, Stx interaction with intestinal epithelial cells and intracellular uptake, and toxin translocation into underlying tissues. Furthermore, it highlights gaps in understanding that need to be addressed by future research.

## 1. Introduction

Enterohaemorrhagic *E. coli* (EHEC) is a major foodborne bacterial pathogen which is responsible for around 1200 cases of gastroenteritis per year in the UK. Although this constitutes only 1–3% of laboratory cases of food poisoning, EHEC infections can lead to severe systemic complications such as haemorrhagic colitis and haemolytic uraemic syndrome (HUS) which can be fatal [1]. Young children and the elderly are particularly at risk, and HUS is the leading cause of acute kidney failure in children in the developed world. At present, there is no specific treatment for HUS, and use of antibiotics remains controversial [2]. HUS is associated with the production of bacterial Shiga toxins (Stx) which are highly cytotoxic to renal microvascular endothelium [3,4]. The glycolipid globotriaosylceramide (Gb3) has been identified as Stx receptor [5].

## 2. Regulation of Stx Production and Release

Shiga toxins from EHEC can be divided into two types, Stx1 and Stx2, which are antigenically different but have the same mode of action [6]. Stx are encoded by the late genes of lambdoid prophages integrated in the bacterial chromosome. Three promotors located upstream of the *stx* genes have been identified: one *stx*-specific promoter (*p_stx_*) and two phage promotors (*p*_R_, *p*_R_’) (reviewed in [7]). Previous studies suggest that *stx1* and *stx2* transcription are regulated differently.

### 2.1. Iron

In most Stx1-producing EHEC strains, *stx1* genes are transcribed from *p_stx1_* which is induced under low iron concentrations by release of Fur-mediated repression [8]. As transcription from *p_stx1_* does not involve expression of the late phage lysis genes, induction of *stx1* expression does not result in bacterial lysis and Stx1 remains within the bacterial cell where it mainly localises in the periplasm [9,10,11].

### 2.2. Phage Lytic Cycle

In contrast to *stx1*, transcription of *stx2* genes is highly dependent on induction of the phage lytic cycle as it is mainly governed by the late phage promoter *p*_R_’ [12]. As a result, *stx2* expression is induced by DNA-damaging agents, such as UV radiation, mitomycin C and certain antibiotics, the use of which is contraindicated during EHEC infection because of enhanced Stx production [9,13,14]. DNA damage results in a bacterial SOS response and activation of RecA which in turn leads to autocleavage of the phage repressor cI and initiates sequential transcription of early and late phage genes. Early genes are responsible for replication of the phage genome thereby increasing the number of *stx2* gene copies within the bacterial cell. Expression of late genes leads to production of Stx, bacterial lysis and toxin release [15,16]. The strong association between *stx2* expression and the phage lytic cycle likely explains why Stx2 is mainly found in supernatants rather than lysates of bacterial cultures [6,10,17]. Increased release of Stx2 compared with Stx1 might contribute to the high HUS risk that has been associated with Stx2-producing EHEC [18,19]: as EHEC are non-invasive and do not cross the intestinal epithelium and cause systemic infection, it is likely that HUS is caused by Stx released into the gut lumen which subsequently gets access to the bloodstream.

### 2.3. Outer Membrane Vesicles

So far, no bacterial secretion system has been identified for Stx, and phage-induced bacterial lysis is assumed to be the main mechanism of toxin release into the environment. However, outer membrane vesicles (OMV), which are naturally shed from the outer membrane of gram-negative bacteria, are gaining increased recognition as delivery vehicles for bacterial virulence factors and toxins (reviewed by [20,21]). Interestingly, it has been demonstrated that OMV from EHEC broth cultures contain Stx1 and Stx2 [22,23]. OMV are formed during bacterial infection and can be internalised by eukaryotic cells as has been reported for toxin delivery by enterotoxigenic *E. coli* [24]. It remains to be investigated whether Stx release in OMV plays a relevant role in Stx delivery across intestinal epithelium and development of HUS.

### 2.4. Intestinal Environment

Regulation of Stx expression has been investigated extensively in bacterial broth cultures but it remains unknown how toxin production is regulated in the complex environment of the human gut. Prominent features of the intestinal milieu include low oxygen levels, the presence of a mucus layer with commensal microflora, and polarised intestinal epithelial cells (IEC).

#### 2.4.1. Oxygen

Studies on the influence of oxygen levels on bacterial virulence gene expression are rare but have recently gained increased attention as the involvement of global regulators of metabolism in pathogenesis is becoming more evident (reviewed in [25]). In the case of EHEC, studies on steady-state chemostat cultures have demonstrated that low oxygen levels *per se* do not affect Stx1 or Stx2 production but lead to increased EHEC host cell adherence [26]. It can be speculated that the latter indirectly leads to higher Stx levels in the gut but this needs to be studied in appropriate infection models.

#### 2.4.2. Microbiota

Infections in mice and *in vitro* studies have demonstrated an inhibitory effect of probiotic bacteria, such as *Lactobacillus* and *Bifidobacterium*, on Stx1 and Stx2 production, and this has been attributed to low pH caused by high levels of acetate production by these bacteria [27,28]. Similarly, secreted factors from human microbiota, in particular from predominant *Bacteroides thetaiotamicron*, inhibit Stx2 production by repression of the phage lytic cyle [29]. The importance of the microflora on Stx levels in the gut is further underlined by a study showing that Stx2 phage infection of susceptible commensal *E. coli* leads to increased Stx2 production [30]. Interestingly, subsequent screening of human intestinal *E. coli* isolates revealed that most strains were resistant to phage infection. However, the rate of about 10% of susceptible isolates corresponds remarkably well with the HUS risk associated with EHEC infection, leading to the hypothesis that HUS susceptibility may be influenced by the intestinal microbiota [30].

#### 2.4.3. Host Cells

Recent *in vitro* studies using a novel Stx luciferase reporter system have demonstrated induced Stx1 and Stx2 transcription by IEC contact [17]. On the other hand, physiological levels of nitric oxide produced by activated IEC inhibit the bacterial SOS response and Stx2 production [31]. While this aspect of the innate immune response to EHEC infection appears to confer a protective effect against Stx-mediated damage, hydrogen peroxide production by human neutrophils has been shown to have the opposite effect and exacerbate Stx2 production [32].

EHEC colonises the follicle-associated epithelium of small intestinal Peyer’s patches *ex vivo* and is therefore likely to come into contact with underlying macrophages [33]. It has been demonstrated that EHEC can survive and multiply in human macrophages for up to 24 h, and enhanced expression of Stx1 and Stx2 was observed during this time [17,34]. Interestingly, both Stx types inhibited EHEC macrophage uptake but promoted intracellular survival [34].

Taken together, these studies indicate that the intestinal environment affects Stx production and release during EHEC infection, and further research in this area will be important to gain a better understanding about the early events leading to HUS pathogenesis.

## 3. Gb3 Expression, Stx Uptake and Intracellular Trafficking

Stx binding and internalisation have been investigated extensively using a wide range of host cells. Although most of the studies have been performed on Stx1 and Stx from *Shigella dysenteriae* type 1 which differs from Stx1 by a single amino acid, it is generally accepted that Stx2 follows the same intracellular transport route and has the same mode of action. It has emerged that Stx cytotoxicity is strongly associated with the amount of Gb3 expressed on the cell surface [35,36], Gb3 density, and association with cholesterol-containing lipid rafts [37,38,39]. In addition, differential Gb3 isoform expression with respect to fatty acid chain length, hydroxylation, and degree of saturation has been shown to affect Stx binding and intracellular transport [40,41,42].

### 3.1. Stx Transport in Sensitive Cells

**Figure 1:**
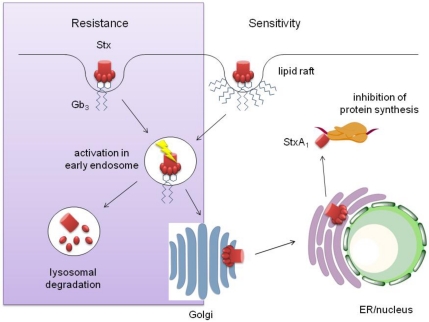
Intracellular Stx transport in resistant and sensitive cells.

Shiga toxins have an AB_5_ structure with one enzymatically active A subunit non-covalently linked to a pentamer of B subunits responsible for Gb3 binding [43]. After binding of Stx to lipid raft-associated Gb3, Stx is internalised into early endosomes and then travels further to the trans-Golgi network (Figure 1). During this early stage, the Stx A subunit is cleaved by furin into an active A1 subunit and an A2 fragment associated with the B pentamer [44]. StxA1 still remains linked to StxA2-B via a disulfide bond and the activated toxin complex is transported via the retrograde pathway to the Golgi apparatus, endoplasmic reticulum (ER) and nuclear membrane [45]. After reduction of the disulfide bond in the ER, StxA1 is retro-translocated into the cytoplasm where it binds to the large ribosomal subunit and inhibits protein synthesis by cleaving off a single adenine residue from the 28S rRNA [46]. While inhibition of protein synthesis in itself does not kill the host cell, it triggers a ribotoxic stress response which ultimately leads to apoptosis [47].

### 3.2. Stx Transport in Resistant Cells

Retrograde Stx transport to the ER and nucleus has been linked to cytotoxicity as toxin trafficking is different in Stx-resistant cells (Figure 1). In macrophages and dendritic cells, Gb3 is not associated with lipid rafts and Stx is transported along the endosomal/lysosomal pathway and ultimately degraded in lysosomes [38]. A similar mechanism of Stx inactivation has also been demonstrated for bovine lower crypt IEC which bind Stx1 but are resistant to cytotoxicity [48]. Cattle are a natural reservoir of EHEC which harmlessly colonise the distal colon [49]. Resistance of bovine intestinal epithelium to Stx and the absence of Gb3 on endothelium most likely contribute to the asymptomatic status of cattle during EHEC infection [50].

Apart from lysosomal degradation, Stx transport in resistant cells such as epidermoid carcinoma, astrocytoma, and ovarian carcinoma cells has been reported to be arrested in the Golgi apparatus. Cytotoxicity can be induced by addition of the differentiation agent butyrate which restores retrograde transport to the ER/nucleus [42,51]. Both studies suggest that this effect is mediated via alteration of Gb3 fatty acid chain length.

### 3.3. Stx Interaction with Human IEC and Intracellular Transport

Human intestinal epithelium represents the first point of contact of released Stx with the host and furthermore acts as a barrier by preventing toxin access to the systemic circulation. Interestingly, normal human intestinal epithelium does not express Gb3 or any other Stx receptor [52,53,54,55]. In contrast, Gb3 expression on colonic epithelium is associated with malignancy and metastasis, and the potential use of Stx in cancer therapy is currently under extensive investigation [56,57,58]. 

Accordingly, most commonly used human colon carcinoma cell lines (Caco-2, HT-29, and HCT-8) express Gb3 on the cell surface and are susceptible to Stx to a variable degree [54,59,60]. Treatment with butyrate has been reported to promote Gb3 expression and Stx sensitivity in Caco-2A and HT-29 cells, indicating that Gb3 expression is regulated by cell maturation [59]. In contrast to these villus-like cell lines, crypt-like colon carcinoma-derived T84 cells do virtually not express Gb3 in the fully differentiated state and are resistant to Stx-induced inhibition of protein synthesis and apoptosis [54,59]. Surprisingly, Stx1 and Stx2 are internalised by T84 cells despite the lack of Gb3 and activated toxin is transported to the ER [54,61]. It still remains to be determined why retrograde toxin transport does not result in T84 cytotoxicity, but it could be speculated that StxA1 release from the ER into the cytoplasm is disrupted. This is supported by recent studies which suggest that retro-translocation is the rate-limiting step in Stx cytotoxicity as only 4% of StxA1 is released into the cytoplasm [62].

Stx interaction with normal (as opposed to cancerous) IEC has been studied by *in vitro* organ culture of human intestinal mucosal biopsies, and results have indicated that Stx2 but not Stx1 causes damage to crypt epithelial cells [54]. As Stx access was not restricted to the mucosal biopsy surface, no conclusions could be drawn as to whether damage was caused by direct Stx-epithelial interaction or indirectly via effects on cells in the underlying lamina propria [54]. Interestingly, recent studies have demonstrated a direct effect of Stx1 on IEC by enhancing galectin-3 secretion and thereby causing mistargeting of absorptive brush border proteins [63]. Notably, this effect could have an implication in EHEC-induced diarrhoea. In addition, it has been shown that Paneth cells, which are key players in the small intestinal innate immune defense, express Gb3 and bind Stx1 and Stx2, and the functional role of this interaction needs to be determined [64].

## 4. Stx Translocation across Intestinal Epithelium

Systemic complications during EHEC infection are caused by Stx and it is assumed that Stx reaches its target sites (kidney and central nervous system) via the bloodstream [3]. Free Stx has not been detected in sera of HUS patients so far, but Stx binding to neutrophils has been demonstrated [65]. After Stx is released into the gut lumen it has to traverse the intestinal epithelium which forms a tight barrier to prevent entry of luminal contents into underlying tissue. The mechanism of this process remains unclear as there are no appropriate animal models available to study this aspect of EHEC infection. Unlike human intestinal epithelium which lacks Gb3 on its surface, Gb3 is expressed by IEC in the mouse distal colon [66]. In rabbits, intestinal epithelial Gb3 expression rapidly increases with age [67]. Human intestinal xenografts in mice have been recently used in EHEC and Stx studies and might provide a valuable model system in the future [55,68].

**Figure 2:**
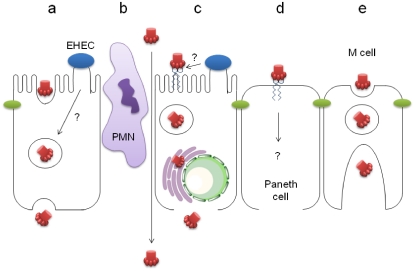
Potential routes of Stx translocation across human intestinal epithelium. (**a**) Gb3-independent transcytosis, possibly enhanced by EHEC infection; (**b**) Paracellular transport during neutrophil (PMN) transmigration; (**c**) Induction of Gb3 expression by EHEC infection, retrograde transport and Stx release after cell death; (**d**) Gb3-dependent translocation by Paneth cells; (**e**) Transcytosis by M cells.

EHEC-induced gastroenteritis is accompanied by severe inflammation and mucosal damage in the caecum and ascending colon [69]. Therefore, it is likely that Stx leaks through damaged epithelium at later stages of infection. However, there are various possible routes of Stx translocation at earlier phases of infection which are described below (Figure 2).

### 4.1. Stx Transcytosis by Gb3-Negative IEC

Earlier studies have demonstrated translocation of purified Stx1 and Stx2 across polarised monolayers of Stx-resistant Caco-2A and T84 colon carcinoma cells. Neither cell viability nor epithelial barrier function was compromised indicating a transcellular pathway of translocation for both Stx types [70,71]. However, differences in translocation rates and directionality, effects of cellular drugs, and competition experiments indicate that Stx1 and Stx2 transcytose IEC by different pathways [71,72]. Transcytosis of Stx1 was further confirmed by immunoelectron microscopy which detected intracellular toxin in association with endosomes, Golgi, ER, and the nuclear membrane, but showed no association with tight junctions [61]. Stx localisation in these cellular compartments is in agreement with retrograde transport in colon carcinoma cells and could be part of the translocation process. However, use of brefeldin A which disrupts the Golgi apparatus, showed no effect on Stx1 or Stx2 translocation indicating that transcytosis is independent of retrograde transport [71]. This is in agreement with more recent studies showing that Stx1 translocation by T84 cells is an unsaturable, Gb3-independent process which involves actin turnover [73]. These criteria apply to the process of macropinocytosis, and it has recently been demonstrated that induced Src kinase-mediated macropinocytosis enhances Stx1 uptake and translocation. Interestingly, results from this study have also shown that EHEC infection can trigger Src kinase activation, thereby suggesting a promoting role of infection in toxin translocation [74].

### 4.2. Paracellular Stx Translocation

EHEC infection causes redistribution of intercellular tight junction proteins such as occludin and ZO-1, and thereby leads to decreased epithelial barrier function. These changes are initiated by type III secretion of the EHEC effector proteins EspF and EspF_U_ into the host cell which trigger subsequent signal transduction events [75,76]. EHEC-mediated disruption of tight junctions may provide a means for Stx to cross the epithelial barrier and this hypothesis has been tested by infecting polarised T84 cells with Stx-negative EHEC to decrease barrier function and subsequently applying Stx1. No significant difference in Stx translocation rate was observed compared with cells treated with purified toxin alone indicating that tight junctions of infected cells still retain their sieving function for larger molecules such as Stx [61].

Another mechanism whereby disruption of tight junctions is likely to occur during infection is by transmigrating neutrophils. Recruitment of neutrophils to infected mucosa is a prominent feature observed during EHEC diarrhoea, and elevated neutrophil levels in stools indicate neutrophil transmigration from the mucosa into the gut lumen [77,78,79,80]. This event has been simulated *in vitro* by co-incubating polarised T84 monolayers with neutrophils on the basal side and inducing neutrophil transmigration by adding a chemoattractant to the apical side. During the process of transmigration, epithelial barrier function was diminished and apical to basal Stx1 and Stx2 translocation was enhanced. Importantly, the authors demonstrated induced neutrophil transmigration by EHEC infection indicating that this process might be relevant in the *in vivo* situation [72].

### 4.3. Stx Uptake by Gb3-Positive IEC

Previous reports have demonstrated that Gb3 expression and Stx1 and Stx2 cytotoxicity on Gb3-positive endothelial cells can be enhanced by treatment with butyrate, pro-inflammatory cytokines or bacterial lipopolysaccharide (LPS) [81,82,83,84]. Similarly, butyrate treatment of Gb3-positive colon carcinoma cells results in increased Stx1 susceptibility due to enhanced Gb3 expression, but this does not affect Gb3-negative T84 cells [59]. The effect of bacterial LPS on Gb3 expression on human IEC has not been tested yet, but it could be speculated that EHEC infection induces epithelial Gb3 expression (by LPS or other bacterial factors) and thereby leads to toxin uptake, retrograde transport and cell death. In addition, EHEC infection results in severe inflammation of the intestinal mucosa which might lead to Gb3 induction on IEC. This hypothesis has been tested by comparing Stx1/Stx2 binding to normal and inflamed intestinal mucosa. No difference in Stx binding was detected between the two groups and no expression of epithelial Gb3 was observed in inflamed samples [64]. However, Gb3 expression and Stx binding to Paneth cells was observed in 30–60% of samples and it remains to be investigated whether Paneth cells might provide an entry site for Stx into underlying tissues [64].

### 4.4. Stx Translocation by M Cells

*In vitro* organ culture of human intestinal biopsies has shown that EHEC adheres predominantly to the follicle-associated epithelium of small intestinal Peyer’s patches [33]. This area is particularly rich in M (microfold) cells which are specialised in uptake and translocation of antigens from the intestinal lumen to underlying antigen-presenting cells. Because of their transcytotic activity, M cells are often exploited by intestinal pathogens for invasion into underlying tissues and have also been implicated in translocation of bacterial toxins [85,86]. It is tempting to speculate that EHEC and/or Stx can breach the epithelial barrier via M cells and future research in this area is needed to test this possibility.

## 5. Conclusions

So far, only little information is available on the regulation of Stx expression and toxin transport in the human gut. Further research is necessary to address the influence of environmental factors in the human intestine on Stx production and release. In addition, appropriate experimental model systems need to be developed which are based on native human IEC rather than cancer-derived cell lines to reflect Gb3 expression patterns in healthy tissues. As it is becoming more evident that EHEC infection might influence Stx uptake and translocation by IEC, future studies should be performed in the context of infection rather than using purified Stx alone. Ultimately, a better understanding of Stx-related events in the human gut is necessary and important as it will lead to the development of early intervention strategies against HUS.
